# MnO_2_/rGO/CNTs Framework as a Sulfur Host for High-Performance Li-S Batteries

**DOI:** 10.3390/molecules25081989

**Published:** 2020-04-23

**Authors:** Wei Dong, Lingqiang Meng, Xiaodong Hong, Sizhe Liu, Ding Shen, Yingkai Xia, Shaobin Yang

**Affiliations:** 1 College of Material Science and Engineering, Liaoning Technical University, Fuxin 123000, China; lgddongwei@163.com (W.D.); mlqzjy@163.com (L.M.); lntutg@126.com (D.S.); 2 College of Mechanical Engineering, Liaoning Technical University, Fuxin 123000, China; lntu001@yeah.net; 3 College of Mining, Liaoning Technical University, Fuxin 123000, China; xiayingkai200719@126.com

**Keywords:** lithium sulfur battery, α-MnO_2_, carbon nanotubes, composites, cathode material

## Abstract

Lithium-sulfur batteries are very promising next-generation energy storage batteries due to their high theoretical specific capacity. However, the shuttle effect of lithium-sulfur batteries is one of the important bottlenecks that limits its rapid development. Herein, physical and chemical dual adsorption of lithium polysulfides are achieved by designing a novel framework structure consisting of MnO_2_, reduced graphene oxide (rGO), and carbon nanotubes (CNTs). The framework-structure composite of MnO_2_/rGO/CNTs is prepared by a simple hydrothermal method. The framework exhibits a uniform and abundant mesoporous structure (concentrating in ~12 nm). MnO_2_ is an α phase structure and the α-MnO_2_ also has a significant effect on the adsorption of lithium polysulfides. The rGO and CNTs provide a good physical adsorption interaction and good electronic conductivity for the dissolved polysulfides. As a result, the MnO_2_/rGO/CNTs/S cathode delivered a high initial capacity of 1201 mAh g^−1^ at 0.2 C. The average capacities were 916 mAh g^−1^, 736 mAh g^−1^, and 547 mAh g^−1^ at the current densities of 0.5 C, 1 C, and 2 C, respectively. In addition, when tested at 0.5 C, the MnO_2_/rGO/CNTs/S exhibited a high initial capacity of 1010 mAh g^−1^ and achieved 780 mAh g^−1^ after 200 cycles, with a low capacity decay rate of 0.11% per cycle. This framework-structure composite provides a simple way to improve the electrochemical performance of Li-S batteries.

## 1. Introduction

With the development of science and technology, the most widely used lithium-ion battery has gradually failed to meet the needs of technological development due to its limited capacity [1,2]. High energy density is the key point of battery development [3,4]. In order to further improve the capacity of secondary batteries, researchers turned their research attention to other battery systems with high energy densities. Among them, the Li-S battery system has attracted widespread attention due to its high theoretical capacity of 1672 mAh g^−1^, which is much more than that of Li-ion batteries (~372 mAh g^−1^ based on graphite anodes) [5]. Therefore, the Li-S batteries have become one of the key research focuses for the next generation battery system, with great developmental potential [6,7].

The typical discharge reaction of Li-S batteries is *16Li + S_8_*→*8Li_2_S* [8]. During this reaction process, a series of soluble intermediates Li_2_S_x_ (Li_2_S_4_, Li_2_S_6_, and Li_2_S_8_) are produced, which can dissolve in the electrolyte and move between the positive and negative electrodes. This phenomenon is often called the shuttle effect, causing the negative electrode to corrode and the capacity to be significantly reduced. The shuttle effect is one of the main factors limiting the development of Li-S batteries. Physical adsorption and chemical adsorption are two commonly used methods to limit the shuttling of lithium polysulfides in lithium-sulfur batteries. Various porous materials are adopted to block the polysulfides in the pores by physical adsorption. The chemical adsorption can be achieved by the chemical bonding interaction between the polar sulfur hosts and lithium polysulfides. Herein, a carbon-based porous composite containing MnO_2_ is designed as a sulfur host to achieve the dual adsorption interaction to improve the electrochemical performance of lithium-sulfur batteries. The developed host material was melted with sulfur at 155 °C and served as a cathode in lithium-sulfur batteries. Physical adsorption materials mainly include carbon materials, such as porous carbon [9], graphene [10], carbon nanotubes [11], and carbon fibers [12], and rely on the large specific surface area [13]. Chemical adsorption materials generally have higher polarity and can form chemical bonds with polysulfides. Therefore, polar molecules can be used to inhibit polysulfide dissolution by producing dipole attraction to polysulfide molecules. Compared with carbon materials without polarity, the interaction between polar materials and polysulfide molecules is greatly enhanced. Among the polar materials, metal oxides are important adsorption materials, including TiO_2_ [14], SiO_2_ [15], Ti_4_O_7_ [16], Al_2_O_3_ [17], MgO [18], Co_9_S_8_ [19], MoS_2_ [20], etc. These materials have a significant effect on the improvement of cycle performance. However, the problem of polar materials is the poor electronic conductivity. Both sulfur and the intermediate products of lithium polysulfides have poor electronic conductivity. Therefore, the sulfur host must have a good electronic conductivity to maintain the electron transfer. In addition, from the EIS test, good conductivity of the sulfur host will induce a low internal resistance and fast reaction kinetics of sulfur. Therefore, the electronic conductivity of the sulfur host is the key factor for deciding the cell performance. In order to improve the electrochemical performance, a regular conductive matrix is urgently needed.

MnO_2_ is a highly polar material that is relatively cheap and easily available [21,22]. It has been widely researched in lithium ion batteries [23,24]. There are four phases of MnO_2_, α, β, γ, and δ. Recently, MnO_2_ was proposed as a sulfur host in Li-S batteries. For instance, Liang et al. [25] designed a polysulfide mediator δ-MnO_2_ for Li-S batteries that displayed an initial capacity of ~1300 mA h g^−1^ at 0.05 C. At the same time, when the S content was 75%, it still had strong chemical adsorption performance. Chen et al. [21] developed a hollow nitrogen-doped micropore-rich carbon (NMRC)@MnO_2_ nanocomposite framework as the sulfur host for advanced Li-S batteries. The polar MnO_2_ nanosheets and doped nitrogen atoms present a strong chemisorption to lithium polysulfides. Li et al. [26] found that the outstanding performance might be attributed to a combination of adsorption, catalytic properties of MnO_2_, and the conductivity network of carbon. Fernando et al. [27] synthesized a framework consisting of tailored γ-MnO_2_ nanorods and an activated biomass-derived carbon (AC/MnO_2_) as the sulfur host. The initial discharge capacity of the AC/MnO_2_/S composite was 874 mAh g^−1^ at 0.1 C, much higher than that of the AC/S composite (692 mAh g^−1^). A β-MnO_2_ framework for Li-S batteries was also prepared and exhibited good cycling stability [28], because the β-MnO_2_/S offers the advantage of rapid Li^+^ transport through the mesoporous walls of β-MnO_2_, when compared with that of the mesoporous carbon/S composites.

The α-MnO_2_ has a three-dimensional open channel structure that can accommodate metal atoms. It has been widely applied in catalysts, and has been proven to have the highest catalytic activity among the four structures (α, β, γ, and δ-MnO_2_) [29,30]. Based on this, α-MnO_2_ may also be used as an adsorbent for polysulfides. However, there are few studies on α-MnO_2_ in Li-S batteries. The interactions between the polysulfides and α-MnO_2_ should be further explored. Meanwhile, the combination of insulating α-MnO_2_ with a conductive matrix is also necessary for improving its performance [31]. Herein, physical and chemical dual adsorption of lithium polysulfides is proposed by designing a novel MnO_2_/rGO/CNTs framework structure consisting of α-MnO_2_ nanorods, reduced graphene oxide (rGO), and carbon nanotubes (CNTs), which is prepared by a simple hydrothermal method. This novel framework structure could provide a simple way to confine the shuttling of dissolved polysulfides in Li-S batteries.

## 2. Results and Discussions

### 2.1. Microstructure and Composition

**Figure 1:**
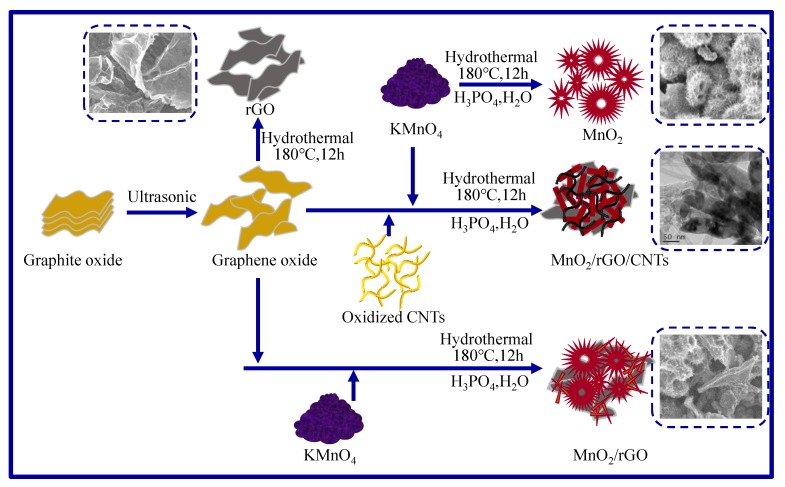
The schematic synthesis processes of MnO_2_, MnO_2_/rGO, and MnO_2_/rGO/CNTs. rGO: reduced graphene oxide; CNTs: carbon nanotubes.

The preparation process is schematically illustrated in Figure 1. The pure MnO_2_ was obtained only by the direct hydrothermal reaction. The reduced graphene oxide (rGO) was obtained through two steps of ultrasonic and hydrothermal reactions from graphite oxide. Serving as a template, rGO was used to generate MnO_2_ in situ, then, the composite of rGO/MnO_2_ was obtained after the addition of KMnO_4_ and H_3_PO_4_. One half of the GO was replaced by oxidized CNTs and the other conditions were maintained with the rGO/MnO_2_. The MnO_2_/rGO/CNTs framework was easily obtained.

**Figure 2:**
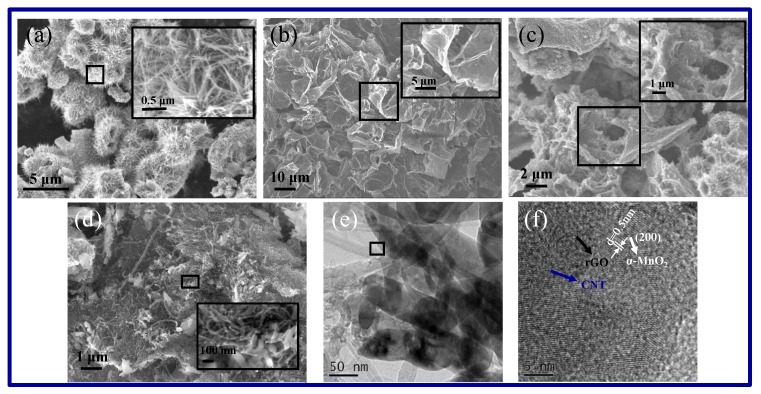
SEM images of MnO_2_ (**a**), rGO (**b**), MnO_2_/rGO (**c**), and MnO_2_/rGO/CNTs (**d**), HRTEM images of MnO_2_/rGO/CNTs (**e, f**).

In order to further characterize the microstructure of the samples, a scanning electron microscope (SEM) was used, as shown in Figure 2. It can be observed from Figure 2 that the MnO_2_ is composed of many hollow urchin-like pompons with diameters of about 3~6 μm. The surface of the urchin-like pompons consists of many fine nanorods with a diameter of about 20 nm. The growth mechanism of the urchin-like MnO_2_ structure can be explained by the Ostwald ripening process [32]. The so-called Ostwald ripening refers to the phenomenon where the particles of the second phase grow up at the cost of the dissolution of the first phase particles, driven by the surface energy difference [33]. Generally, permanganate is thermodynamically unstable and can be easily reduced to produce MnO_2_ (4MnO_4_^-^ + 4H^+^ = 4MnO_2_ + 3O_2_ + 2H_2_O). In an acidic environment or at high temperatures, the reaction occurs spontaneously and rapidly. In the initial stage of the reaction, a large number of manganese dioxide nuclei are generated in a short period of time, and these nuclei are continuously aggregated to form microspheres with a solid core. These microspheres will grow into a one-dimensional MnO_2_ nanorod as a core, and the nanorods grow epitaxially along the surface of the initial microsphere to form an urchin-like nanostructure. From Figure 2b, it can be seen that rGO is composed of many irregular yarns, more like flakes instead of the regular graphite structure, which indicates that the regular structure of graphite was seriously damaged by oxidation, ultrasonic treatment, and subsequent hydrothermal treatment. Figure 2c shows the morphology of the MnO_2_/rGO composite. The urchin-like MnO_2_ is attached to the surface and the defects of rGO. At the same time, the MnO_2_ and rGO are interwoven and combined perfectly, and the composite has abundant big holes. When CNTs were added into the system of MnO_2_ and rGO, the morphology of the composite was changed to some extent, as shown in Figure 2d. The MnO_2_ in the MnO_2_/rGO/CNTs composite was no longer urchin-like, the MnO_2_ short nanorods were combined with CNTs and rGO to form a framework-structure composite, which may be attributed to the nucleation particles of oxidized CNTs. From the HRTEM images of MnO_2_/rGO/CNTs, the MnO_2_ short nanorods (diameter of 50 nm and length of about 200 nm) were compounded with carbon nanotubes (diameter of 20–50 nm) and tulle-like rGO (Figure 2g). Meanwhile, the HRTEM image (Figure 2f) of a single nanorod shows a lattice spacing of 0.5 nm, which corresponds to the (200) crystal plane spacing of α-MnO_2_ [34].

**Figure 3:**
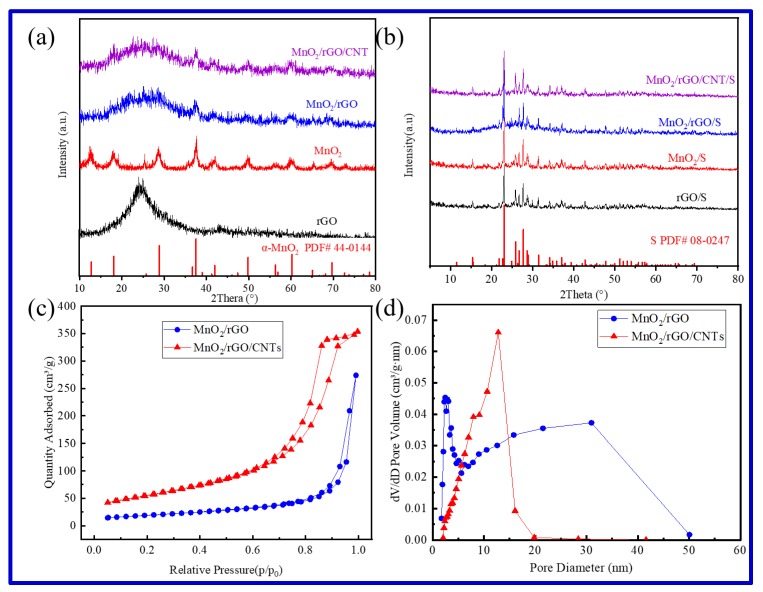
XRD patterns of rGO, MnO_2_, MnO_2_/rGO, and MnO_2_/rGO/CNTs (**a**); XRD patterns of rGO/S, MnO_2_/S, MnO_2_/rGO/S, and MnO_2_/rGO/CNTs/S (**b**); Nitrogen adsorption–desorption isotherms (**c**) and pore size distribution curves (**d**) for MnO_2_/rGO and MnO_2_/rGO/CNTs.

The obtained rGO, MnO_2_, MnO_2_/rGO, and MnO_2_/rGO/CNTs were characterized by X-ray diffraction (XRD), as shown in Figure 3a. The diffraction spectra of the obtained MnO_2_ can be well matched to α-MnO_2_ (JSCD No.44-0144). The intensity of MnO_2_ diffraction peaks are low, and the full width at half maxima (FWHM) is large, which indicates that the grain size of the synthesized MnO_2_ is smaller. The rGO was obtained after ultrasonic and hydrothermal treatment; a broad peak at 24° and a very weak peak at 43° appeared, which were corresponded to the (002) and (101) diffractions of the carbon materials, respectively. However, compared with GO the crystallinity of rGO is greatly reduced. The patterns of the MnO_2_/rGO and MnO_2_/rGO/CNTs are quite similar with two diffraction spectra that could be well indexed to α-MnO_2_ (JSCD No.44-0144), indicating that the MnO_2_ synthesized is α phase. Except for the characteristic diffraction peak of α-MnO_2_, a broad peak at 24° and a very weak peak at 43°, corresponding to the diffractions of the carbon materials, were found, indicating the presence of carbon. The thermal stability was characterized by thermal gravimetric analysis (TGA) in air, as given in Figure S1. The content of MnO_2_ in MnO_2_/rGO and MnO_2_/rGO/CNTs are about 49.6 wt% and 50.8 wt%, respectively. The MnO_2_ content of the two samples is almost the same. The diffraction spectra of rGO/S, MnO_2_/S, MnO_2_/rGO/S, and MnO_2_/rGO/CNTs/S are shown in Figure 3b. The only phase that can be clearly distinguished is S, and neither carbon (rGO and CNTs) nor MnO_2_ were detected, due to their lower content and crystallinity.

Figure 3c,d shows the N_2_ adsorption–desorption isotherm and pore size distribution curves of MnO_2_/rGO and MnO_2_/rGO/CNTs. The N_2_ adsorption–desorption isotherm of MnO_2_/rGO exhibits the type III isotherm and H3 hysteresis loop (Figure 3c), indicating a mesoporous or macroporous structure, which is given in the pore size distribution (Figure 3d). MnO_2_/rGO has a specific surface area of 109 m^2^ g^−1^ and total pore volume of 0.42 cm^3^ g^−1^. The N_2_ adsorption–desorption isotherm of MnO_2_/rGO/CNTs exhibits the type IV isotherm and H1 hysteresis loop (Figure 3c), indicating a uniform mesoporous structure, as given in the pore size distribution (Figure 3d), the pore was concentrated in ~12 nm. The MnO_2_/rGO/CNTs composite has a higher specific surface area of 232 m^2^ g^−1^ and total pore volume of 0.55 cm^3^ g^−1^. These abundant mesopores are crucial for achieving high S loading and confining lithium polysulfides inside the framework of MnO_2_/rGO/CNTs. The porous framework of MnO_2_/rGO/CNTs not only facilitates the transportation of electrolytes, but also provides abundant active sites for the adsorption of lithium polysulfides.

### 2.2. Electrochemical Performance

**Figure 4:**
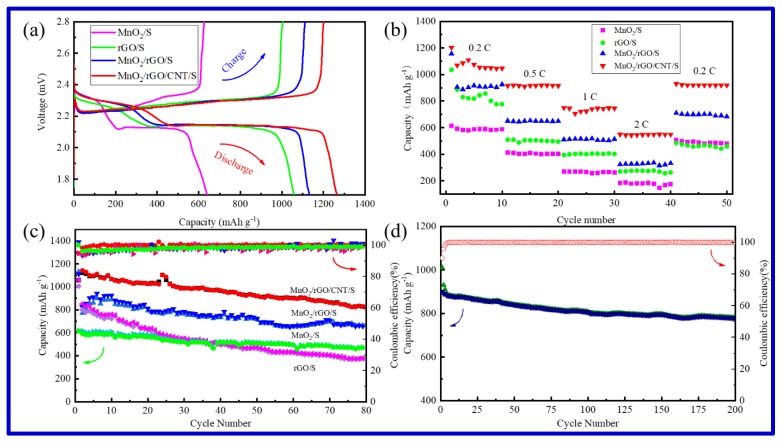
Charge–discharge curves (**a**); rate performance (**b**); cycling performance and coulombic efficiency (**c**) of rGO/S, MnO_2_/S, MnO_2_/rGO/S, and MnO_2_/rGO/CNTs/S; long cycle performance of MnO_2_/rGO/CNTs/S (**d**).

The electrochemical performance of the as-prepared rGO/S, MnO_2_/S, MnO_2_/rGO/S, and MnO_2_/rGO/CNTs/S cathodes were evaluated by galvanostatic charge–discharge measurements, as shown in Figure 4. The charge/discharge curves of the four samples at 0.2 C are presented in Figure 4a. There are two main stages in the discharge curves at about 2.3 V and 2.1 V. The high voltage platform is a short platform ascribed to the formation of long-chain lithium polysulfides (Li_2_S_8_~Li_2_S_4_). The low voltage platform is a long platform ascribed to the formation of short chain polysulfides (Li_2_S_2_ and Li_2_S). The as-prepared rGO/S, MnO_2_/S, MnO_2_/rGO/S, and MnO_2_/rGO/CNTs/S cathodes delivered initial discharge capacities of 1059 mAh g^−1^, 640 mAh g^−1^, 1134 mAh g^−1^, and 1266 mAh g^−1^, respectively. The initial discharge capacities of rGO/S, MnO_2_/rGO/S, and MnO_2_/rGO/CNTs/S samples are not significantly different, indicating the Coulombic efficiencies of these samples are similar. The initial discharge capacity of MnO_2_/S is relatively low, indicating the sulfur utilization rate is low, which may be related to the poor conductivity of MnO_2_ hosts.

The cycling performance of rGO/S, MnO_2_/S, MnO_2_/rGO/S, and MnO_2_/rGO/CNTs/S cathodes are presented in Figure 4b. With the increasing of cycling number, the capacity of the four samples decreased gradually. The capacity of MnO_2_/rGO/CNTs/S remained the highest (826 mAh g^−1^) after 80 cycles at 0.2 C, which is much higher that other samples. It can be seen from the cycling performance of the above materials that the initial discharge capacity of rGO/S is relatively high, but the capacity retention rate is the lowest, indicating that the adsorption of lithium polysulfides is poor. Although the initial capacity of MnO_2_/S is lower, the capacity retention rate is relatively high, which proves to some extent that α-MnO_2_ has a significant effect on the adsorption of lithium polysulfides. The cycle performance of MnO_2_/rGO/S is better than rGO/S and MnO_2_/S, indicating that the combination of MnO_2_ and rGO is beneficial to electrochemical performance. After replacing some rGO with CNTs, the three-dimensional frame structure of MnO_2_/rGO/CNTs is established. The capacity and capacity retention rate of the composite increased significantly, indicating that MnO_2_/rGO/CNTs framework structure effectively improved the electrochemical performance by introducing the conductive CNTs. This is related to the pore size distribution of the three-dimensional sulfur-loaded framework structure and the synergistic effect of rGO and CNTs.

The rate performance of rGO/S, MnO_2_/S, MnO_2_/rGO/S, and MnO_2_/rGO/CNTs/S cathodes are presented in Figure 4c. Among them, MnO_2_/rGO/CNTs/S has the best rated performance. The average capacity was 1080 mAh g^−1^ at 0.2 C, and then decreased to 916 mAh g^−1^ at 0.5 C, 736 mAh g^−1^ at 1 C, and 547 mAh g^−1^ at 2 C. When the current density returned to 0.2 C, the average capacity recovered to 920 mAh g^−1^, which is close to its original capacity, indicating a good rate capability in a wide range. This performance may be related to the better conductivity of the materials. In addition, the MnO_2_/rGO/CNTs/S cathode delivered a high initial capacity of 1010 mAh g^−1^ at 0.5 C and achieved 780 mAh g^−1^ after 200 cycles (Figure 4d), with a high capacity retention of 77.2%. The capacity decay rate is 0.11% per cycle. Moreover, when compared with the reported MnO_2_-containing composite hosts (Table S1), the MnO_2_/rGO/CNTs/S cathode delivers a comparable or even better electrochemical performance [21,27,35,36,37,38,39,40].

**Figure 5:**
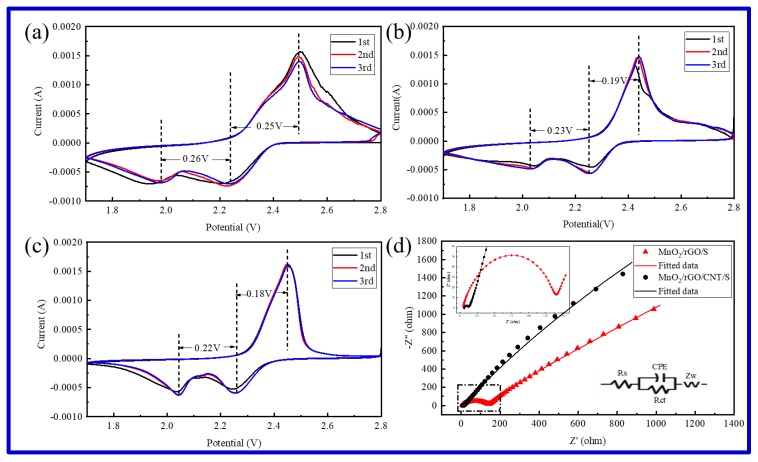
Cyclic voltammograms of rGO/S (**a**), MnO_2_/rGO/S (**b**), and MnO_2_/rGO/CNTs/S (**c**); electrochemical impedance spectroscopy (EIS) curves of MnO_2_/rGO/S and MnO_2_/rGO/CNTs/S (**d**).

Figure 5a–c show the cyclic voltammogram (CV) profiles of rGO/S, MnO_2_/rGO/S, and MnO_2_/rGO/CNTs/S, respectively. According to the literature [41,42], there are two reduction peaks and one oxidation peak in the CV curves of typical Li-S batteries. During the reduction process, two peaks appeared at ~2.2 V and ~2.0 V, corresponding to the conversion of element sulfur to the soluble high-order lithium polysulfides (Li_2_S_x_, 4 ≤ x ≤ 8) and the soluble high-order polysulfides (Li_2_S_x_, 4 ≤ x ≤ 8) to insoluble low-order lithium sulfides (Li_2_S_2_, Li_2_S). During the oxidation reaction, the peak at ~2.4 V is related to the reversible conversion from insoluble low-order lithium sulfides to element S. The rGO/S, MnO_2_/rGO/S, and MnO_2_/rGO/CNTs/S exhibit characteristic redox peaks of typical Li-S batteries. With the increase of cycling number, the peak areas of rGO/S show a significant decrease, indicating that the adsorption effect of polysulfide is not obvious. However, the MnO_2_/rGO/S and MnO_2_/rGO/CNTs/S perform much better. The polarization between the reduction peaks and the oxidation peaks of the rGO/S composite is much larger than that of the MnO_2_/rGO/S and MnO_2_/rGO/CNTs/S cathodes. The sharp redox peaks in the CV curves of MnO_2_/rGO/CNTs/S indicate fast-electrochemical kinetics. In addition, the overlap of redox peak positions demonstrates a good electrochemical reversibility, which is consistent with the cycling performance, indicating that the framework structure of MnO_2_/rGO/CNTs is beneficial to the electrochemical reaction process.

The electrochemical impedance spectroscopy (EIS) plots of MnO_2_/rGO/S and MnO_2_/rGO/CNTs/S investigated after three cycles (at 2.8 V) are given in Figure 5d. The results could be well fitted by an equivalent circuit in Figure 5d. The two curves have a similar feature. They both have an overlapping semicircle in the high and medium frequency ranges, corresponding to the charge-transfer resistance (R_ct_) on the solid/electrolyte interface, and an inclined line at low frequencies, owing to the Warburg impedance (Z_w_) of ion diffusion in the materials. The intersection of the EIS curve in the high frequency region represents the system resistance (R_s_). The fitting data of equivalent circuits are presented in Table 1. The values of MnO_2_/rGO/CNTs/S are much smaller than those of MnO_2_/rGO/S, which indicates that MnO_2_/rGO/CNTs framework effectively promotes the reversible electrochemical reaction by enhancing the conductivity of sulfur hosts.

## 3. Materials and Methods

### 3.1. Preparation of Materials

Synthesis of rGO. First, 11.445 g graphite oxide aqueous solution (GO, prepared by Hummers method, and the concentration of graphite oxide was 1.2 wt%) was sonicated for 10 h and then was put directly into a thermostatic oven (AX-f100, Xiniu Technology company, Beijing, China) at 180 °C for 12 h. The resultant product was freeze-dried and denoted (version, Beijing Biocool Experimental Instrument Co., Ltd., Beijing, China) as rGO.

Synthesis of Urchin-like MnO_2_**.** Urchin-like MnO_2_ was synthesized by a one-step facile method. Briefly, 5.88 g KMnO_4_ was dissolved in 30 mL deionized water to prepare the KMnO_4_ solution. Then, 6.09 g H_3_PO_4_ (85 wt%) was added to the KMnO_4_ solution, and then the mixture was transferred into a thermostatic oven at 180 °C for 12 h. The resulting material was washed with deionized water.

Synthesis of MnO_2_/rGO. A mixed solution containing 5.88 g KMnO_4_ and 6.09 g H_3_PO_4_ were prepared according to the above method. Then, 11.455 g graphite oxide aqueous solution (as above, after sonication for 10 h) was dispersed in the mixed solution by sonication. Then, the mixed solution was transferred into a thermostatic oven at 180 °C for 12 h. The product was washed with deionized water. Finally, the MnO_2_/rGO composite was obtained by the freeze-drying method.

Synthesis of MnO_2_/rGO/CNTs. Multiwalled carbon nanotubes (CNTs, Kelude) were oxidized by the modified Hummers method [43]. In the preparation process of MnO_2_/rGO, one half of GO was replaced by oxidized CNTs, and other conditions were maintained. Finally, MnO_2_/rGO/CNTs was obtained.

The MnO_2_/rGO/CNTs, MnO_2_/rGO/S, MnO_2_/S, and rGO/S composites were prepared by diffusion method. Sulfur powder and MnO_2_/rGO/S, MnO_2_/S, or rGO/S composites were mixed at a mass ratio of 35:65 and then milled, respectively. Subsequently, the mixtures were heated at 155 °C for 12 h and cooled to room temperature. The S content in each sample was about 65%.

### 3.2. Materials Characterization

The structures were characterized by using an X ray diffract meter (XRD) (XRD, XRD-6000, Shimadzu, Tokyo, Japan). The surface morphologies and microstructures were investigated using a scanning electron microscope (SEM) (JSM-7800F, JEOL, Tokyo, Japan) and high-resolution transmission electron microscope (HRTEM) (JEOL JEM-2100F, Tokyo, Japan). The N_2_ adsorption–desorption isotherms and pore size distributions were characterized by using a specific surface and pore size distribution analyzer (Autosorb-iQ).

### 3.3. Electrochemical Measurements

The battery was assembled in an argon filled glove box with a 2032 battery case. The slurry, consisting of 80 wt.% active material, 10 wt.% carbon black, and 10 wt.% polyvinylidene difluoride (PVdF), was smeared uniformly on an aluminum foil (Canrd, ~20 μm), then was dried at 60 °C for 30 min. The samples were transferred into avacuum oven and kept at 60 °C for 8 h. The mass loading on the electrode was about 2.1 mg cm^−2^, and the diameter of the electrode was 1.6 cm. The pure lithium foil was employed as the anode. The separator was Cellgard−2400. The electrolyte was a 1 M lithium bis(trifluoromethanesulfonyl)imide (LiTFSI) solution in DOL and DME (volume ratio 1:1) with 2 wt% LiNO_3_. Galvanostatic charge–discharge tests were completed on a NEWARE test system with a cut-off voltage window of 1.7~2.8 V. Cyclic voltammetry (scanning speed 0.2 mV/s, cut-off voltage window of 1.7~2.8 V) and the electrochemical impedance spectroscopy (from 0.01 Hz to100 kHz, at 5 mV) were measured on a CHI660E electrochemical workstation (CHI660E, Shanghai CH Instrument Co., Ltd., Shanghai, China).

## 4. Conclusions

In summary, we designed and fabricated a framework-structure composite containing α-MnO_2_, reduced graphene oxide (rGO), and carbon nanotubes (CNTs) as a sulfur host by a simple hydrothermal method. The framework exhibits a uniform and abundant mesoporous structure (concentrating in ~12 nm). MnO_2_ has an α phase structure and the α-MnO_2_ also has a significant effect on the adsorption of lithium polysulfides, similar to all other phase structures of MnO_2_. These abundant mesopores are crucial for achieving high S loading and confining lithium polysulfides inside the framework of MnO_2_/rGO/CNTs. The rGO and CNTs provide a good physical adsorption interaction and good electronic conductivity for the active sulfur. The initial capacity of the MnO_2_/rGO/CNTs cathode was 1201 mAh g^−1^. The average capacities at the current densities of 0.5 C, 1 C, and 2 C were 916 mAh g^−1^, 736 mAh g^−1^, and 547 mAh g^−1^, respectively. When tested at 0.5 C, the MnO_2_/rGO/CNTs/S cathode delivered a high initial capacity of 1010 mAh g^−1^, and achieved 780 mAh g^−1^ after 200 cycles, with a high capacity retention of 77.2%. The capacity decay rate was 0.11% per cycle. This framework-structure composite provides a simple way to improve the electrochemical performance of Li-S batteries.

## Figures and Tables

**Table 1:** The EIS data of the samples.

Sample	Rs(Ω)	Rct(Ω)
MnO_2_/rGO/S	5.6	106.1
MnO_2_/rGO/CNTs/S	2.9	22.6

## Supplementary Materials

The following are available online, Figure S1: TGA curves of MnO_2_/rGO and MnO_2_/rGO/CNTs, Table S1: Electrochemical properties of the reported MnO_2_-containing composite hosts for Li-S batteries.

## References

[1] Hong X., Wang R., Liu Y., Fu J., Liang J., Dou S. (2020). Recent advances in chemical adsorption and catalytic conversion materials for Li–S batteries. J. Energy Chem..

[2] Petnikota S., Rotte N.K., Reddy M.V., Srikanth V.V.S.S., Chowdari B.V.R. (2015). MgO-decorated few-layered graphene as an anode for Li-ion batteries. ACS Appl. Mater. Interfaces.

[3] Raić M., Mikac L., Marić I., Štefanić G., Škrabić M., Gotić M., Ivanda M. (2020). Nanostructured silicon as potential anode material for Li-ion batteries. Molecules.

[4] Kulkarni P., Nataraj S.K., Balakrishna R.G., Nagaraju D.H., Reddy M.V. (2017). Nanostructured binary and ternary metal sulfides: Synthesis methods and their application in energy conversion and storage devices. J. Mater. Chem. A.

[5] Appadurai T., Subramaniyam C.M., Kuppusamy R., Karazhanov S., Subramanian B. (2019). Electrochemical Performance of Nitrogen-Doped TiO_2_ Nanotubes as Electrode Material for Supercapacitor and Li-Ion Battery. Molecules.

[6] Rana M., Ahad S.A., Li M., Luo B., Wang L., Gentle I., Knibbe R. (2019). Review on areal capacities and long-term cycling performances of lithium sulfur battery at high sulfur loading. Energy Storage Mater..

[7] Tsao C.H., Hsu C.H., Zhou J.D., Chin C.W., Kuo P.L., Chang C.H. (2018). Vulcanized polymeric cathode material featuring a polyaniline skeleton for high-rate rechargeability and long-cycle stability lithium-sulfur batteries. Electrochim. Acta.

[8] Tao X., Wang J., Liu C., Wang H., Yao H., Zheng G., Seh Z.W., Cai Q., Li W., Zhou G. (2016). Balancing surface adsorption and diffusion of lithium-polysulfides on nonconductive oxides for lithium-sulfur battery design. Nat. Commun..

[9] Fu A., Wang C., Pei F., Cui J., Fang X., Zheng N. (2019). Recent Advances in Hollow Porous Carbon Materials for Lithium-Sulfur Batteries. Small.

[10] Fang R., Chen K., Yin L., Sun Z., Li F., Cheng H.-M. (2019). The Regulating Role of Carbon Nanotubes and Graphene in Lithium-Ion and Lithium-Sulfur Batteries. Adv. Mater..

[11] Garapati M.S., Piriya A.V.S., Sundara R. (2019). Synergy between partially exfoliated carbon nanotubes-sulfur cathode and nitrogen rich dual function interlayer for high performance lithium sulfur battery. Carbon N. Y..

[12] Zhu M., Wang Y., Long L., Fu X., Sui G., Yang X. (2019). An optimal carbon fiber interlayer integrated with bio-based gel polymer electrolyte enabling trapping-diffusion-conversion of polysulfides in lithium-sulfur batteries. Chem. Eng. J..

[13] Zhang L., Wang Y., Niu Z., Chen J. (2019). Advanced nanostructured carbon-based materials for rechargeable lithium-sulfur batteries. Carbon N. Y..

[14] Zha C., Wu D., Zhang T., Wu J., Chen H. (2019). A facile and effective sulfur loading method: Direct drop of liquid Li2S8 on carbon coated TiO2 nanowire arrays as cathode towards commercializing lithium-sulfur battery. Energy Storage Mater..

[15] Liu T., Sun X., Sun S., Niu Q., Liu H., Song W., Cao F., Li X., Ohsaka T., Wu J. (2019). A robust and low-cost biomass carbon fiber@SiO2 interlayer for reliable lithium-sulfur batteries. Electrochim. Acta.

[16] Guo Y., Li J., Pitcheri R., Zhu J., Wen P., Qiu Y. (2019). Electrospun Ti_4_O_7_/C conductive nanofibers as interlayer for lithium-sulfur batteries with ultra long cycle life and high-rate capability. Chem. Eng. J..

[17] Zhu F., Liu J., Zhao H., Li J., Li Q., Xi Y., Liu M., Wang C. (2019). Preparation and Performance of Porous Polyetherimide/Al2O3 Separator for Enhanced Lithium-Sulfur Batteries. Chemelectrochem.

[18] Sun W., Sun X., Peng Q., Wang H., Ge Y., Akhtar N., Huang Y., Wang K. (2019). Nano-MgO/AB decorated separator to suppress shuttle effect of lithium-sulfur battery. Nanoscale Adv..

[19] Lin H., Zhang S., Zhang T., Cao S., Ye H., Yao Q., Zheng G.W., Lee J.Y. (2019). A Cathode-Integrated Sulfur-Deficient Co9S8 Catalytic Interlayer for the Reutilization of “Lost” Polysulfides in Lithium-Sulfur Batteries. ACS Nano.

[20] Chen M., Yin X., Reddy M.V., Adams S. (2015). All-solid-state MoS_2_/Li_6_PS_5_Br/In-Li batteries as a novel type of Li/S battery. J. Mater. Chem. A.

[21] Chen H., Dong W.D., Xia F.J., Zhang Y.J., Yan M., Song J.P., Zou W., Liu Y., Hu Z.Y., Liu J. (2020). Hollow nitrogen-doped carbon/sulfur@MnO_2_ nanocomposite with structural and chemical dual-encapsulation for lithium-sulfur battery. Chem. Eng. J..

[22] Tu S., Zhao X., Cheng M., Sun P., He Y., Xu Y. (2019). Uniform Mesoporous MnO_2_ Nanospheres as a Surface Chemical Adsorption and Physical Confinement Polysulfide Mediator for Lithium-Sulfur Batteries. ACS Appl. Mater. Interfaces.

[23] Nithyadharseni P., Reddy M.V., Fanny H., Adams S., Chowdari B.V.R. (2015). Facile one pot synthesis and Li-cycling properties of MnO_2_. RSC Adv..

[24] Reddy M.V., Subba Rao G.V., Chowdari B.V.R. (2013). Metal oxides and oxysalts as anode materials for Li ion batteries. Chem. Rev..

[25] Tan S., Yang Z., Yuan H., Zhang J., Yang Y., Liu H. (2019). MnO_2_-decorated graphene aerogel with dual-polymer interpenetrating network as an efficient hybrid host for Li-S batteries. J. Alloys Compd..

[26] Xiaoman L., Qinglin Z., Weimin G., Qinghua L. (2019). The catalytic activity of manganese dioxide supported on graphene promoting the electrochemical performance of lithium-sulfur batteries. J. Electroanal. Chem..

[27] Luna-Lama F., Hernández-Rentero C., Caballero A., Morales J. (2018). Biomass-derived carbon/γ-MnO_2_ nanorods/S composites prepared by facile procedures with improved performance for Li/S batteries. Electrochim. Acta.

[28] Wang S., Yang Z., Zhang H., Tan H., Yu J., Wu J. (2013). Mesoporous β-MnO_2_/sulfur composite as cathode material for Li-S batteries. Electrochim. Acta.

[29] Cao Y.L., Yang H.X., Ai X.P., Xiao L.F. (2003). The mechanism of oxygen reduction on MnO_2_-catalyzed air cathode in alkaline solution. J. Electroanal. Chem..

[30] Kalubarme R.S., Cho M.S., Yun K.S., Kim T.S., Park C.J. (2011). Catalytic characteristics of MnO_2_ nanostructures for the O-2 reduction process. Nanotechnology.

[31] Liu X., Huang J.Q., Zhang Q., Mai L. (2017). Nanostructured Metal Oxides and Sulfides for Lithium–Sulfur Batteries. Adv. Mater..

[32] Xiao-Miao F., Zhen-Zhen Y., Ning-Na C. (2014). Synthesis of Hollow Urchin-Like MnO_2_ via a Facile Hydrothermal Method and Its Application in Supercapacitors. Chinese J. Inorg. Chem..

[33] Zeng H.C. (2007). Ostwald ripening: A synthetic approach for hollow nanomaterials. Curr. Nanosci..

[34] Su T., Zhao B., Fan B., Li H., Zhang R. (2019). Enhanced microwave absorption properties of novel hierarchical core-shell delta/alpha MnO_2_ composites. J. Solid State Chem..

[35] Cao K., Liu H., Li Y., Wang Y., Jiao L. (2017). Encapsulating sulfur in δ-MnO_2_ at room temperature for Li-S battery cathode. Energy Storage Mater..

[36] Li Z., Zhang J., Lou X.W. (2015). Hollow Carbon Nanofibers Filled with MnO_2_ Nanosheets as Efficient Sulfur Hosts for Lithium-Sulfur Batteries. Angew. Chemie Int. Ed..

[37] Dang R., Ma X., Liu J., Chen M., Zhang Y., Luo J. (2018). Mesoporous MnO_2_ fibers as an efficient bifunctional absorber for high-performance lithium-sulfur batteries. Int. J. Hydrogen Energy.

[38] Chen M., Lu Q., Jiang S., Huang C., Wang X., Wu B., Xiang K., Wu Y. (2018). MnO_2_ nanosheets grown on the internal/external surface of N-doped hollow porous carbon nanospheres as the sulfur host of advanced lithium-sulfur batteries. Chem. Eng. J..

[39] Huang X., Shi K., Yang J., Mao G., Chen J. (2017). MnO_2_-GO double-shelled sulfur (S@MnO_2_@GO) as a cathode for Li-S batteries with improved rate capability and cyclic performance. J. Power Sources.

[40] Ling B., Chen A., Liu W., Liu K., Hu H., Zhang J. (2018). Simply and rapidly synthesized composites of MnO_2_ nanosheets anchoring on carbon nanotubes as efficient sulfur hosts for Li-S batteries. Mater. Lett..

[41] Kim A.-Y., Kim M.K., Kim J.Y., Wen Y., Gu L., Dao V.-D., Choi H.-S., Byun D., Lee J.K. (2017). Ordered SnO nanoparticles in MWCNT as a functional host material for high-rate lithium-sulfur battery cathode. Nano Res..

[42] Xu C., Wu Y., Zhao X., Wang X., Du G., Zhang J., Tu J. (2015). Sulfur/three-dimensional graphene composite for high performance lithium-sulfur batteries. J. Power Sources.

[43] Liang Y., Wang H., Diao P., Chang W., Hong G., Li Y., Gong M., Xie L., Zhou J., Wang J. (2012). Oxygen Reduction Electrocatalyst Based on Strongly Coupled Cobalt Oxide Nanocrystals and Carbon Nanotubes. J. Am. Chem. Soc..

