# Supplementary material for: MnO2/rGO/CNTs Framework as a Sulfur Host for High-Performance Li-S Batteries
Source: Molecules. 2020 Apr 23;25(8):1989. doi: 10.3390/molecules25081989 (PMC7221920; doi:10.3390/molecules25081989)
Supplement: Supplementary file 1 [file molecules-25-01989-s001.pdf]

MnO_2/rGO/CNTs Framework as a Sulfur Host for High-Performance Li-S Batteries

Wei Dong [1], Lingqiang Meng [1], Xiaodong Hong [1,*], Sizhe Liu [2], Ding Shen [1], Yingkai Xia [3] and Shaobin Yang [1,*]

[1] College of Material Science and Engineering, Liaoning Technical University, Fuxin 123000, China; lgddongwei@163.com (W.D.); mlqzjy@163.com (L.M.)
[2] College of Mechanical Engineering, Liaoning Technical University, Fuxin 123000, China; lntu001@yeah.net
[3] College of Mining, Liaoning Technical University, Fuxin 123000, China; xiayingkai200719@126.com
* Correspondence: hongxiaodong@lntu.edu.cn (X.H.); yangshaobin@lntu.edu.cn (S.Y.)

Academic Editors: Vivek Kumar and Charles C. Chusuei
Received: 30 March 2020; Accepted: 22 April 2020; Published: 23 April 2020

The thermal stability was characterized by thermal gravimetric analysis (TGA) in air, shown in Fig. S1. The content of MnO_2 in MnO_2/rGO and MnO_2/rGO/CNTs are about 49.6 wt% and 50.8 wt%, respectively.

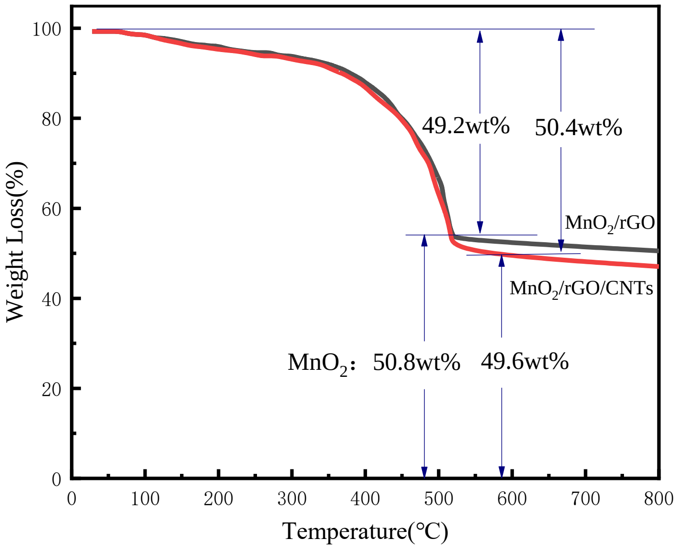

Figure S1. TGA curves of MnO_2/rGO and MnO_2/rGO/CNTs.

Table S1. Electrochemical properties of the reported MnO_2-containing composite hosts for Li-S batteries.

Material	FCC[a] (mAh g^{-1}(A/g))	CAC[b] (mAh g^{-1})	CD[c] ($cycle^{-1}$)	Ref
AC/MnO2/S@ ht	874(0.1 C)	555(100)	0.36%	27
S@MnO2	~1200(0.1)	760(200)	0.18%	35
NMRC/S@MnO_2	1144(0.2C)	1023(200)	0.053%	21
MnO2@HCF/S	900(0.5C)	662(300)	0.088%	36
MOF/S	1015 (0.1)	815 (200)	0.098%	37
NHCSs@MnO_2/S	1283(0.2C)	847(500)	0.067%	38
S@MnO2@GO	603(0.35C)	261(400)	0.22%	39
CNTs/MnO2-S.	1056 (0.5)	841(100)	0.20%	40
This work	1010.7 (0.5)	780.3(200)	0.11%	-

[a] First Charge Capacity; [b] Capacity After (x)Cycles; [c] Capacity Decay.
